# Prognostic value of perioperative changes in the prognostic nutritional index in patients with surgically resected non-small cell lung cancer

**DOI:** 10.1007/s00595-024-02847-5

**Published:** 2024-05-03

**Authors:** Kazuki Hayasaka, Hirotsugu Notsuda, Ken Onodera, Tatsuaki Watanabe, Yui Watanabe, Takaya Suzuki, Takashi Hirama, Hisashi Oishi, Hiromichi Niikawa, Yoshinori Okada

**Affiliations:** 1 Department of Thoracic Surgery, Tohoku University Hospital, Sendai, Japan; 2 Department of Thoracic Surgery, Institute of Development, Aging and Cancer, Tohoku University, 4-1 Seiryocho, Aobaku, Sendai, 980-8575 Japan

**Keywords:** Immunonutrition, Prognostic nutritional index, Lung cancer, Surgery, Prognosis

## Abstract

**Purpose:**

This single-institution retrospective cohort study was conducted to assess the prognostic significance of perioperative changes in the prognostic nutritional index (PNI) in patients who underwent surgery for non-small cell lung cancer (NSCLC).

**Methods:**

Clinicopathological data were collected from 441 patients who underwent lobectomy for NSCLC between 2010 and 2016.The PNI ratio (postoperative PNI/preoperative PNI) was used as an indicator of perioperative PNI changes. Prognostic differences were investigated based on PNI ratios.

**Results:**

The optimal cut-off value of the PNI ratio for overall survival (OS) was set at 0.88 using a receiver operating characteristic curve. The PNI ratio was inversely related to a high smoking index, interstitial lung disease, and postoperative pulmonary complications. The 5-year OS rates for the high vs. low PNI ratio groups were 88.2% vs. 68.5%, respectively (hazard ratio [HR]: 3.04, 95% confidence interval [CI]: 1.90–4.86). Multivariable analysis revealed that a low PNI ratio was significantly associated with poor prognosis (HR: 2.94, 95% CI: 1.77–4.87). The PNI ratio was a more sensitive indicator than postoperative PNI status alone for identifying patients at high risk of mortality, particularly those with non-lung cancer causes.

**Conclusion:**

The perioperative PNI change is a significant prognostic factor for patients with NSCLC.

**Supplementary Information:**

The online version contains supplementary material available at 10.1007/s00595-024-02847-5.

## Introduction

Immunonutritional status has emerged as a significant prognostic indicator for patients with various types of malignant tumors and is believed to reflect their overall condition [1–6]. The prognostic nutritional index (PNI) is an immunonutritional index measured by serum albumin levels and the peripheral total lymphocyte count (TLC) [7]. The preoperative PNI (pre-PNI) has been identified as a prognostic factor for patients with surgically resected non-small cell lung cancer (NSCLC) [8–13]. These relationships have also been observed in patients with pathological stage I NSCLC [9, 10], those receiving adjuvant platinum-based chemotherapy [13], and geriatric patients [8]. Postoperative PNI (post-PNI) has also been reported as a prognostic factor [14].

Despite this knowledge, the effect of alterations in the host immunonutritional status during the perioperative period on the prognosis of patients with surgically resected NSCLC remains unclear; yet, assessing perioperative changes may reflect the patient’s general condition more accurately than single-point evaluations before or after surgery. A previous report documented the negative impact of postoperative decline in serum albumin levels on the prognosis of patients with early-stage NSCLC [15]. However, to our knowledge, this is the first study to explore the association between perioperative changes in the PNI and prognosis after NSCLC surgery. Given that the PNI reflects both immune and nutritional status, it is plausible that it may provide a more accurate indication of prognosis than nutritional status alone.

The objective of this retrospective cohort study, conducted at a single institution, was to investigate whether a decrease in the PNI during the perioperative period was associated with poor prognosis in patients with surgically resected NSCLC. Additionally, we investigated whether perioperative changes in the PNI are a more accurate prognostic factor than pre- and post-PNI values.

## Methods

Ethical approval for this study was obtained from the Ethics Committee of Tohoku University Hospital (IRB number 2021-1-912-1) and the requirement for informed consent from individual patients was waived.

### Study design and patient selection

**Fig. 1:**
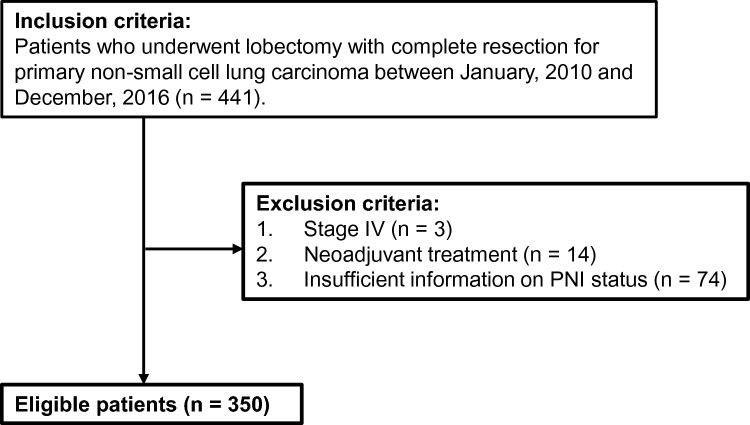
Patient selection algorithm

This was a retrospective cohort study conducted at a single institution. For analysis, data were obtained from our institution’s NSCLC database, which includes clinicopathological and surgical factors, laboratory data, adjuvant treatment, postoperative complications, and prognosis. The clinical and pathological TNM classifications followed the 8th edition of the UICC for International Cancer Control staging manual [16]. We reviewed the records of consecutive patients who underwent complete resection with lobectomy at the Tohoku University Hospital between January, 2010 and December, 2016, resulting in an initial sample size of 441 patients. Patients who received neoadjuvant treatment, those who had stage IV disease, and those with incomplete PNI data were excluded, resulting in a final sample size of 350 (Fig. 1). No nutritional interventions were performed during the hospital stay.

### Data collection

Preoperative and postoperative blood tests were conducted 1 month before and 1 month after surgery, respectively. PNI was calculated using the equation: 10 × serum albumin (g/dL) + 0.005 × TLC (cells/mm^3^) [7]. We used the PNI ratio, calculated as the post-PNI value divided by the pre-PNI value, as a metric to evaluate changes in the perioperative PNI. In terms of adjuvant chemotherapy, we adhered to the clinical recommendations outlined by the Japan Lung Cancer Society, which recommend tegafur-uracil for patients with stage IB disease and platinum-based chemotherapy for patients with stage II or III disease [17].

The severity of postoperative cardiovascular and pulmonary complications was assessed based on the Clavien-Dindo classification system [18, 19] or the Common Terminology Criteria for Adverse Events (CTCAE) v5.0. Complications of grade II or higher in the Clavien-Dindo classification system or grade 2 or higher in the CTCAE were considered in this study. Postoperative pulmonary complications included air leakage, atelectasis, bronchial obstruction, hypoxemia, pleural effusion, pneumonia, empyema, chylothorax, exacerbation of interstitial lung disease, and chronic obstructive pulmonary disease. Prolonged air leakage exceeding 7 days following surgery, requiring additional drainage or pleural adhesive therapy constituted manifestations of air leakage. Grade II/2 or higher bronchial obstruction was that necessitating medical or endoscopic interventions for symptoms indicative of bronchial obstruction. Similarly, grade II/2 or higher pleural effusion was that necessitating medical or surgical interventions (such as thoracic drainage or pleural adhesive therapy) due to symptoms indicative of pleural effusion. Postoperative cardiovascular complications included atrial fibrillation, thrombosis, and heart failure.

Postoperative surveillance involved monthly outpatient visits for the first 2 months and then every 3–6 months for 5 years. Postoperative recurrence was assessed by clinical examination, computed tomography (CT), positron emission tomography/CT, or magnetic resonance imaging. The date of recurrence was defined as the date of diagnosis based on imaging or histological confirmation. Histological confirmation of recurrence was not required if it was clinically evident.

### Statistical analysis

Receiver operating characteristic (ROC) curve analysis was performed to identify the optimal cut-off value for the PNI ratio to distinguish patients who died during the study period from those who survived. Wilcoxon and Fisher’s exact tests were used to compare background characteristics between the patient groups for continuous and categorical variables, respectively. The primary outcome was overall survival (OS), and the secondary outcomes were recurrence rate and recurrence-free survival (RFS). OS considered death from any cause as an event, whereas RFS considered disease recurrence after surgery or death from any cause as an event. For patients without adverse events, the date of the last follow-up was used for censoring. The Kaplan–Meier method was used to establish OS and RFS curves, which were evaluated using the log-rank test. Univariable and multivariable Cox proportional hazards regression analyses were conducted to identify the prognostic factors for OS. Statistical analyses were performed using the JMP® Pro 16.1.0 (SAS Institute Inc., Cary, NC, USA). All statistical tests were two-sided, and a *P* value <0.05 was considered significant.

## Results

### Patient characteristics

Initially, we calculated the optimal cut-off of the PNI ratio for OS using ROC curve analysis, which was 0.88 (Supplemental Fig. 1). Based on this cut-off, patients were stratified into two groups: those with a high PNI ratio (PNI ratio > 0.88; *n* = 261) and those with a low PNI ratio (PNI ratio ≤ 0.88; *n* = 89). As shown in Table 1, a low PNI ratio was significantly associated with a high Brinkman index and interstitial lung disease as comorbidities. Although no significant differences were observed between the two groups in terms of other preoperative, surgical, or pathological factors, patients with a low PNI ratio had significantly higher incidences of specific postoperative pulmonary complications, including prolonged air leakage, atelectasis, bronchial obstruction, hypoxemia, and pleural effusion (Table 1; Supplemental Table 1).

**Table 1:** Association between perioperative changes of the prognostic nutritional index and clinicopathological variables

Variables	PNI ratio		*P*
	Low	High	
	*n* = 89	*n* = 261	
Sex			
Female (%)	37 (41.6)	123 (47.1)	0.39
Age			
Years, median (IQR)	70 (64–74)	68 (63–75)	0.50
Smoking history			
Former or current (%)	61 (68.5)	166 (63.6)	0.44
Brinkman index			
Median (IQR)	660 (0–1000)	330 (0–800)	0.04
BMI			
kg/m^2^, median (IQR)	22.8 (20.2–25.0)	23.1 (20.7–25.0)	0.68
Performance status*			
0 (%)	80 (89.9)	234 (89.7)	1.00
Past medical history			
+ (%)			
Hypertension	24 (27.0)	75 (28.7)	0.79
Ischemic heart disease	9 (10.1)	22 (8.4)	0.67
Arrhythmia	3 (3.4)	14 (5.4)	0.58
Cerebrovascular disease	6 (6.7)	14 (5.4)	0.60
Diabetes mellitus	13 (14.6)	32 (12.3)	0.58
Bronchial asthma	1 (1.1)	4 (1.5)	1.00
Obstructive pulmonary disease	4 (4.5)	5 (1.9)	0.24
Interstitial lung disease	8 (9.0)	7 (2.7)	0.03
Malignant diseases	28 (31.5)	74 (28.4)	0.59
FVC			
%, Median (IQR)	107 (97–116)	109 (98–121)	0.20
FEV1/FVC ratio			
%, Median (IQR)	74 (66–80)	76 (69–81)	0.07
DLCO			
%, Median (IQR)	102 (85–121)	106 (89–121)	0.30
Preoperative blood test			
AST			
U/L, Median (IQR)	22 (17–28)	20 (18–24)	0.29
ALT			
U/L, Median (IQR)	18 (14–25)	16 (13–25)	0.17
eGFR			
mL/min, Median (IQR)	71 (60–78)	72 (63–82)	0.20
Total protein			
g/dL, Median (IQR)	7.1 (6.8–7.5)	7.0 (6.6–7.2)	
Albumin			
g/dL, Median (IQR)	4.1 (3.9–4.4)	4.0 (3.7–4.2)	<0.01
Lymphocyte			
/mm^3^, Median (IQR)	1780 (1370–2180)	1600 (1350–2010)	0.01
PNI			
Median (IQR)	50.6 (47.4–53.1)	48.6 (45.1–51.2)	<0.01
CRP			
mg/dL, Median (IQR)	0.1 (0.1–0.2)	0.1 (0.1–0.2)	0.61
CEA			
ng/dL, Median (IQR)	3.1 (2.0–7.5)	2.9 (1.9–4.9)	0.14
CT pattern			
Pure solid tumor (%)	33 (37.1)	106 (40.6)	0.78
SUVmax			
Median (IQR)	4.5 (2.4–8.3)	4.7 (2.3–8.6)	0.97
Clinical stage			
0	1 (1.1)	5 (1.9)	0.17
IA/IB	43 (48.3)/15 (16.9)	122 (46.7)/66 (25.3)	
II	22 (24.7)	45 (17.2)	
III	8 (9.0)	23 (8.8)	
Mediastinal lymph node dissection			
+ (%)	69 (77.5)	217 (83.1)	0.27
Operative duration			
min, median (IQR)	232 (191–274)	221 (180–270)	0.21
Blood loss			
mL, Median (IQR)	59 (15–140)	38 (11–95)	0.08
Epidural anesthesia			
+ (%)	77 (86.5)	224 (85.8)	
Histologic type			
(%)			
Adenocarcinoma	65 (73.0)	201 (77.0)	0.39
Squamous cell carcinoma	17 (19.1)	43 (16.5)	
Others	7 (7.9)	17 (6.5)	
Pathological invasive tumor size			
cm, Median (IQR)	2.3 (1.6–3.3)	2.2 (1.5–3.0)	0.40
Lymph node involvement			
+ (%)	21 (23.6)	48 (18.4)	0.28
Pathological stage			
(%)			
0	0 (0.0)	1 (0.4)	0.23
IA/IB	38 (42.7)/19 (21.4)	137 (52.5)/48 (18.4)	
IIA/IIB	7 (7.9)/11 (12.4)	8 (3.1)/41 (15.7)	
IIIA/IIIB	12 (13.5)/2 (2.3)	22 (8.4)/4 (1.5)	
Pleural invasion			
+ (%)	29 (32.6)	78 (29.9)	0.69
Lymphatic permeation			
+ (%)	27 (30.3)	84 (32.2)	0.79
Vascular invasion			
+ (%)	43 (48.3)	108 (41.4)	0.27
Adjuvant chemotherapy			
+ (%)	19 (21.8)	65 (26.4)	0.47
Postoperative pulmonary complications			
+ (%)	35 (39.3)	34 (13.0)	<0.01
Postoperative cardiovascular complications			
+ (%)	7 (7.9)	22 (8.4)	1.00
Preoperative blood test			
Total protein			
g/dL, Median (IQR)	6.6 (6.3–6.9)	7.1 (6.8–7.3)	<0.01
Albumin			
g/dL, Median (IQR)	3.3 (3.1–3.6)	3.9 (3.7–4.1)	<0.01
Lymphocyte			
/mm^3^, Median (IQR)	1330 (1025–1675)	1700 (1420–2125)	<0.01
PNI			
Median (IQR)	40.2 (36.2–43.2)	47.6 (44.7–50.9)	<0.01
CRP			
mg/dL, Median (IQR)	1.1 (0.4–2.1)	0.2 (0.1–0.5)	<0.01

*PNI* prognostic nutritional index, *BMI* body mass index, *FVC* forced volume capacity, *FEV* forced expiratory volume, *DLCO* diffusing capacity of the lung for carbon monoxide, *AST* aspartate aminotransferase, *ALT* alanine aminotransferase, *CRP* C-reactive protein, *CEA* carcinoembryonic antigen, *CT* computed tomography, *SUV* standardized uptake value, *IQR* interquartile range

### PNI ratio in relation to postoperative prognosis

The median length of follow-up for the censored cases among the 350 eligible patients was 73 months (interquartile range: 61–90). Two patients died within 90 days of surgery: one from lung cancer and one from an unrelated illness. During the follow-up period, 70 patients (20.0%) died and 80 (22.9%) suffered recurrence. The recurrence rate was not significantly different between the high and low PNI ratio groups (*P* = 0.47); however, patients with a low PNI ratio had a significantly higher mortality rate, particularly related to other diseases, than those with a high PNI ratio (all-cause mortality:37.1% vs. 14.2%, *P* < 0.01; death from other diseases: 23.6% vs. 4.6%, *P* < 0.01).

**Fig. 2:**
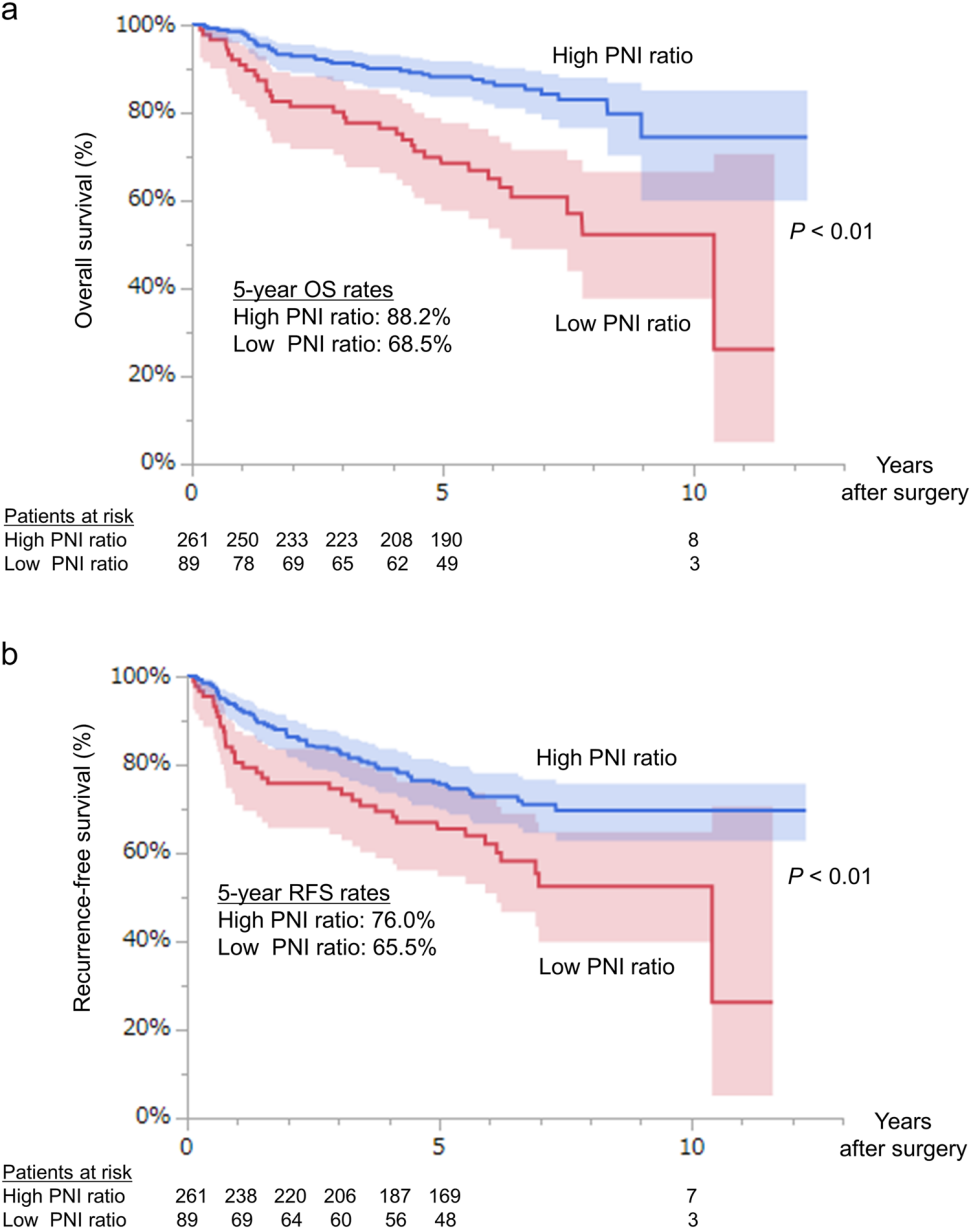
Kaplan–Meier curves for **a** postoperative overall survival (OS) and **b** recurrence-free survival (RFS) according to the prognostic nutrition index (PNI) ratio: high PNI ratio (> 0.88 [blue line]) or low PNI ratio (≤0.88 [red line]). *P* values were calculated by log-rank tests

The 5-year OS and RFS rates in the high PNI ratio group were 88.2% and 76.0%, respectively, whereas those in the low PNI ratio group were 68.5% and 65.5%, respectively (Fig. 2a, b). Both OS and RFS were significantly shorter in the low PNI ratio group than in the high PNI ratio group (both *P* < 0.01). Stratified analysis according to the pathological stage and postoperative pulmonary complications showed similar patterns (Supplemental Fig. 2a–d).

In the multivariable analysis, patients with advanced-stage NSCLC, lymphatic permeation, and low PNI ratio had a significantly worse prognosis, with a hazard ratio of 2.94 (95% confidence interval = 1.77–4.87), than those with high PNI ratio (Table 2).

**Table 2:** Univariable and multivariable analyses of factors associated with overall survival

Variable	Univariable analysis			Multivariable analysis		*P*
	HR	95% CI	*P*	HR	95% CI	
Age						
≥70/<70 years old	1.29	0.80–2.06	0.29	1.23	0.75–2.02	0.41
Sex						
Male/female	2.65	1.56–4.51	<0.01	1.81	0.92–3.59	0.09
Smoking history						
Ex- or current/never	2.02	1.15–3.52	0.01	1.11	0.54–2.31	0.77
Preoperative PNI						
Per 1 unit increase	1.00	0.95–1.05	0.97	–	–	–
Histologic type						
Non-AD/AD	2.60	1.61–4.20	<0.01	1.58	0.90–2.78	0.11
Pathological stage						
II or III/0 or I	4.33	2.68–7.00	<0.01	2.49	1.36–4.56	<0.01
Pleural invasion						
Yes/no	2.42	1.51–3.87	<0.01	1.50	0.89–2.52	0.13
Lymphatic permeation						
Yes/no	3.21	1.99–5.17	<0.01	1.92	1.05–3.53	0.04
Vascular invasion						
Yes/no	4.35	2.57–7.37	<0.01	1.51	0.74–3.08	0.25
Adjuvant therapy						
No/yes	0.76	0.45–1.27	0.29	1.66	0.92–3.00	0.09
Postoperative PNI						
Per 1 unit increase	0.94	0.91–0.98	<0.01	–	–	–
PNI ratio						
Per 1 unit increase	0.06	0.00–0.43	<0.01	–	–	–
<0.88 (low)/ ≥ 0.88 (high)	3.04	1.90–4.86	<0.01	2.94	1.77–4.87	<0.01
Postoperative pulmonary complications						
Yes/no	2.10	1.26–3.51	<0.01	1.06	0.60–1.87	0.85

*HR* hazard ratio, *CI* confidence interval, *PNI* prognostic nutritional index, *AD* adenocarcinoma

### Comparison of PNI ratio and pre- and postoperative PNI status

**Fig. 3:**
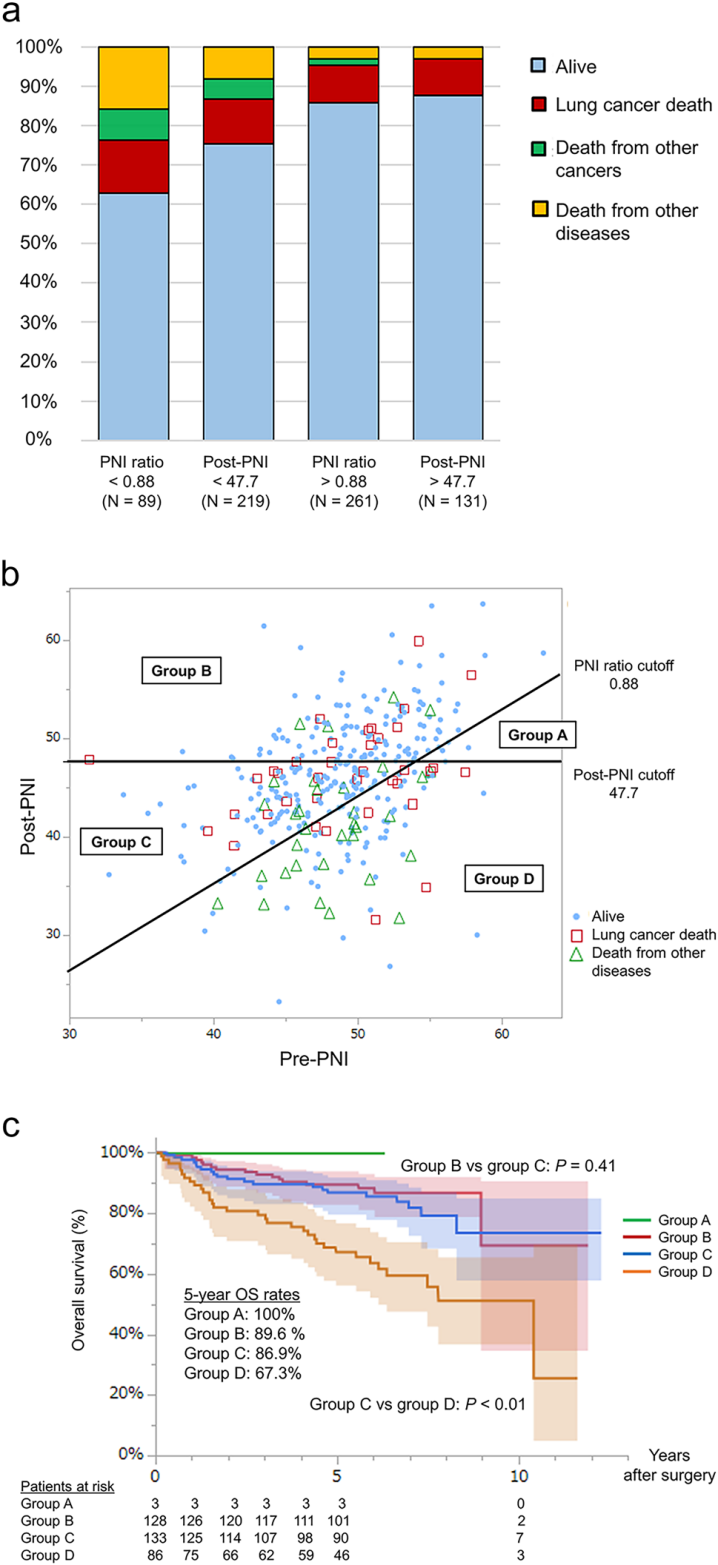
**a** Different impacts of post-PNI status and PNI ratio on mortality and cause of death. **b** Scatter plot showing the association between the preoperative prognostic nutritional index (pre-PNI) and the postoperative PNI (post-PNI) and prognosis. **c** Kaplan–Meier curves for overall survival (OS) according to the postoperative prognostic nutritional index (post-PNI) and PNI ratio: high post-PNI and low PNI ratio (group A [green line]); high post-PNI and high PNI ratio (group B [red line]); low post-PNI and high PNI ratio (group B [blue line]); or low post-PNI and low PNI ratio (group D [orange line]). The *P* values were obtained using log-rank tests

To identify the most sensitive prognostic factors, we assessed the optimal cut-off values for pre- and postoperative PNI using ROC curves. The cut-off values were 48.3 and 47.7, and the area under the curve (AUC) in predicting OS was 0.50 and 0.60, respectively (Supplemental Fig. 3a, b). Notably, the AUC for the post-PNI was equivalent to that of the PNI ratio. We also compared the impact of the post-PNI and PNI ratio on mortality and found that patients with a low PNI ratio had a higher incidence of death, particularly from non-lung cancer, than those with a low post-PNI ratio (Fig. 3a).

For further analysis, we visualized the association between PIN status and prognosis using a scatter plot (Fig. 3b). The patients were stratified into four groups: Group A (high post-PNI and low PNI ratio; *n* = 3), group B (high post-PNI and high PNI ratio; *n* = 128), group C (low post-PNI and high PNI ratio; *n* = 133), and group D (low post-PNI and low PNI ratio; *n* = 86). Among patients with a low post-PNI, a high PNI ratio (group C) was significantly associated with longer OS than a low PNI ratio (group D), and the 5-year OS rates in the high- and low-PNI ratio groups were 86.9% and 67.3%, respectively (*P* < 0.01). Additionally, the 5-year OS of patients in group C was comparable to that of patients with high post-PNI and high PNI ratios (group B) (86.9% vs. 89.6%, *P* = 0.41) (Fig. 3c).

## Discussion

The findings of our study highlight the importance of perioperative changes in the PNI ratio as a negative prognostic factor for patients undergoing surgical resection for NSCLC. A lower PNI ratio was associated with a high smoking index, interstitial lung disease, and postoperative pulmonary complications. Moreover, the PNI ratio was found to be a more sensitive indicator than the post-PNI status alone for identifying patients at high risk of mortality, particularly from non-lung cancer causes.

Hayasaka et al. reported that lung cancer patients with low pre-PNI and low post-PNI tended to have a worse prognosis [14], but the challenge regarding the definition of pre-PNI and post-PNI in that study lies in its population-based classification rather than by individualized assessment. The correlation between perioperative PNI decline in an individual and its association with prognosis remains ambiguous. Therefore, in this study, we focused on the perioperative PNI decline at an individual level. It was also important to identify a parameter that could more precisely and equitably reflect the association between PNI alteration and mortality: the “absolute difference of peri-PNI change” versus the “PNI ratio”. Patients with higher pre-PNI levels might have greater immunonutritional reserve capacity than those with lower pre-PNI levels, complicating prognosis evaluation solely based on absolute peri-PNI changes. Conversely, we posit that utilizing the PNI ratio could alleviate this challenge, potentially enabling a more accurate and equitable comparison in prognosis assessments.

Although previous studies have explored the prognostic impact of the PNI in relation to cancer-specific survival [10, 14], few have investigated its association with non-cancer-specific survival. In our study, a low PNI ratio was significantly associated with a higher risk of death from non-lung cancers. Kamigaichi et al. reported that the postoperative PNI was significantly higher in their segmentectomy group than in their lobectomy group [20]. This finding may help explain the results of a randomized trial that compared the surgical outcomes between lobectomy and segmentectomy for NSCLC, and identified better survival after segmentectomy than after lobectomy, and demonstrated that fewer patients in the segmentectomy arm died of non-lung cancer diseases [21]. Although the underlying mechanisms by which the PNI affects prognosis after NSCLC surgery remain unclear, these reports support the idea that the PNI, as a reflection of the immunonutritional capacity of patients to endure surgical stress, may provide insights into non-cancer-specific survival. Despite the lack of detailed data on the specific causes of death from non-lung cancer diseases, the association between a low PNI ratio, a high smoking index, and interstitial lung disease implies that cardiovascular and lung diseases may contribute to increased mortality of these patients. These findings underscore the importance of managing comorbidities such as cardiovascular and lung diseases through smoking cessation, good management of hypertension, diabetes mellitus, dyslipidemia and hyperuricemia, and implementing suitable respiratory exercise therapy along with intensive postoperative follow-up after lung cancer surgery.

Our study revealed a significant association between a low PNI ratio and postoperative pulmonary complications. Postoperative prolonged inflammation and complications, such as air leakage, atelectasis, pneumonia, and pleural effusion, can lead to hypoalbuminemia and subsequently, lower post-PNI levels. Previous studies have found that postoperative pulmonary complications contribute independently to a poor prognosis for patients with NSCLC [22, 23]. These facts raise the question of whether the PNI is an intermediate factor in respiratory complications. However, in the present study, the multivariable Cox proportional hazards model for OS showed that postoperative pulmonary complications were not a significant prognostic factor. Among patients without postoperative pulmonary complications, a low PNI ratio was significantly associated with a poor prognosis. These results suggest that the PNI ratio is a prognostic factor independent of pulmonary complications.

Furthermore, our study demonstrated that the PNI ratio is a more sensitive indicator than post-PNI status alone for identifying high-risk patients. In this study, an AUC analysis was conducted for pre-PNI, post-PNI, and PNI ratio to ascertain their sensitivity as prognostic factors for predicting OS. Our findings indicated that pre-PNI exhibited lower sensitivity, whereas post-PNI showed comparable sensitivity to the PNI ratio. Among patients with a low post-PNI, we identified two potentially distinct groups: a stable low group (with no decline in PNI ratio; group C in this study) and a declining group (with a decline in PNI ratio; group D). Our results confirmed the prognostic differences between the two groups, emphasizing the utility of the PNI ratio in risk stratification. Notably, this study provides a cut-off value for perioperative PNI changes that can be used in daily medical practice to estimate mortality risk.

Perioperative nutritional intervention may be crucial to relieve the decline in perioperative host immunonutritional status. Preoperative enteral nutrition programs can effectively prevent the reduction in serum albumin and TLC during the perioperative period [24, 25]. Ongoing randomized controlled trials will provide further insights into the effectiveness of enteral nutrition combined with accelerated rehabilitation for improving immunonutritional indicators after surgery [26].

We acknowledge the limitations of this study. First, it was a retrospective cohort study conducted at a single institution, which may limit the generalizability of the findings. Further validation studies are needed to establish the reliable cut-off value of PNI ratio. Second, we did not assess other immunonutritional statuses such as the Controlling Nutritional Status (CONUT) score or the Glasgow Prognostic Score (GPS), which have been reported as prognostic factors in patients with NSCLC undergoing surgical resection [27, 28]. However, direct comparison of the perioperative changes in the PNI, CONUT score, and GPS is challenging because the nature of each of these variables is different. Future studies incorporating multiple immunonutritional indicators are warranted to assess their prognostic value comprehensively.

## Conclusion

Our study demonstrated the significance of perioperative PNI ratio changes as a negative prognostic factor in patients undergoing surgical resection for NSCLC. The PNI ratio outperformed the post-PNI status alone for identifying patients with a high mortality risk, particularly for non-lung cancer causes of death. Managing comorbidities and implementing perioperative nutritional interventions may help to improve the immunonutritional status and outcomes of these patients.

### Supplementary Information

Below is the link to the electronic supplementary material.

Supplementary file1 (DOCX 15 KB)

Supplementary file2 (DOCX 918 KB)
