# Supplementary material for: Prognostic value of perioperative changes in the prognostic nutritional index in patients with surgically resected non-small cell lung cancer
Source: Surg Today. 2024 May 3;54(9):1031–40. doi: 10.1007/s00595-024-02847-5 (PMC11341629; doi:10.1007/s00595-024-02847-5)
Supplement: Supplementary file 1 — Supplementary file1 (DOCX 15 KB) [file 595_2024_2847_MOESM1_ESM.docx]

**Supplemental Table 1.**

Association between perioperative changes of the prognostic nutritional index and postoperative cardiovascular and pulmonary complications

|  | PNI ratio | |  |
| --- | --- | --- | --- |
|  | Low | High |  |
| Postoperative complications, n (%) | n = 89 | n = 261 | *p* |
| Cardiovascular complications | 7 (7.9) | 22 (8.4) | 1.00 |
| Atrial fibrillation | 4 (4.5) | 20 (7.7) | 0.27 |
| Thrombosis | 2 (2.3) | 2 (0.8) | 0.47 |
| Heart failure | 1 (1.1) | 0 (0.0) | 0.25 |
| Pulmonary complications | 35 (39.3) | 34 (13.0) | < 0.01 |
| Prolonged air leak | 12 (13.5) | 16 (6.1) | 0.04 |
| Atelectasis, bronchial obstruction | 8 (9.0) | 7 (2.7) | 0.03 |
| Hypoxemia | 6 (6.7) | 5 (1.9) | 0.03 |
| Pleural effusion | 4 (4.5) | 2 (0.8) | 0.04 |
| Pneumonia | 2 (2.3) | 3 (1.2) | 0.60 |
| Empyema | 2 (2.3) | 0 (0.0) | 0.06 |
| Exacerbation of ILD | 2 (2.3) | 0 (0.0) | 0.06 |
| Chylothorax | 1 (1.1) | 0 (0.0) | 0.25 |
| Exacerbation of COPD | 0 (0.0) | 1 (0.4) | 1.00 |
| PNI: Prognostic nutritional index, ILD: Interstitial lung disease, COPD: Chronic obstructive pulmonary disease | | | |
