# Supplementary material for: Prognostic value of perioperative changes in the prognostic nutritional index in patients with surgically resected non-small cell lung cancer
Source: Surg Today. 2024 May 3;54(9):1031–40. doi: 10.1007/s00595-024-02847-5 (PMC11341629; doi:10.1007/s00595-024-02847-5)

**Supplemental Figure 1.** A receiver operating characteristic curve was used to determine the optimal cut-off value of the prognostic nutritional index (PNI) ratio for overall survival. AUC: Area under the curve


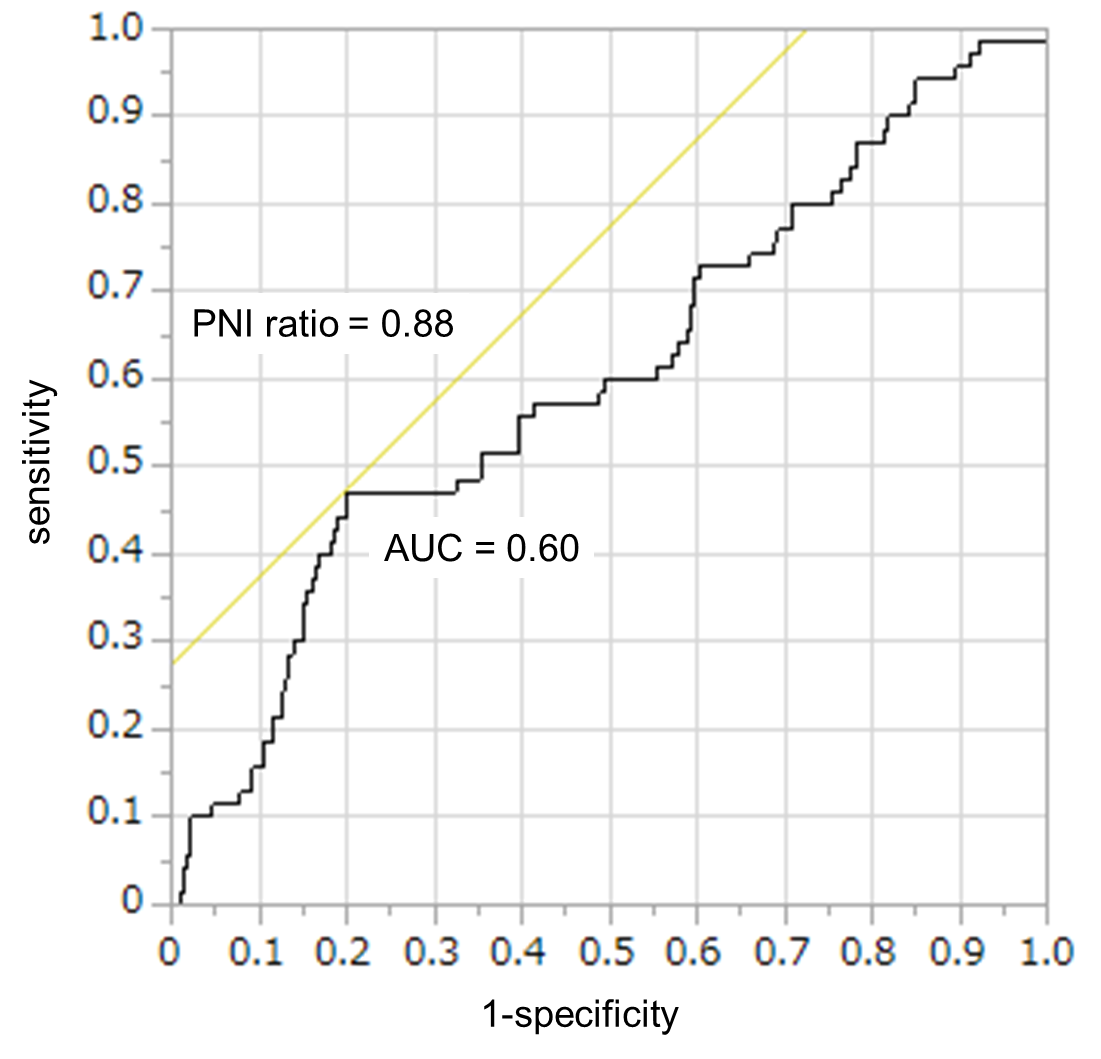


**Supplemental Figure 2.** Kaplan–Meier curves for overall survival (OS) stratified by (a) pathological stage I, (b) stage II or III, and (c) presence or (d) absence of postoperative pulmonary complications, according to the prognostic nutritional index (PNI) ratio: low PNI ratio (red line) or high PNI ratio (blue line). P-values were obtained using log-rank tests.

(a)


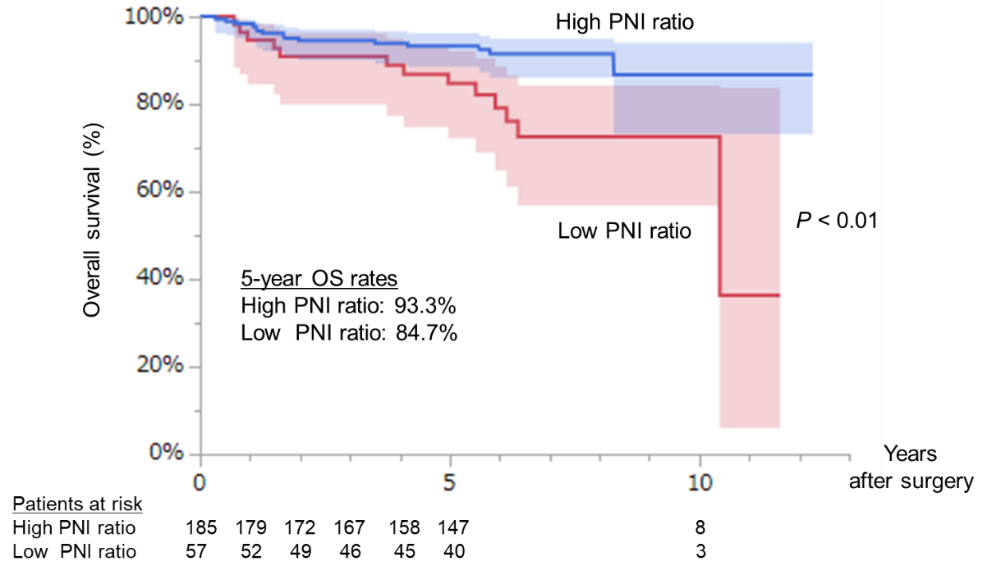


(b)


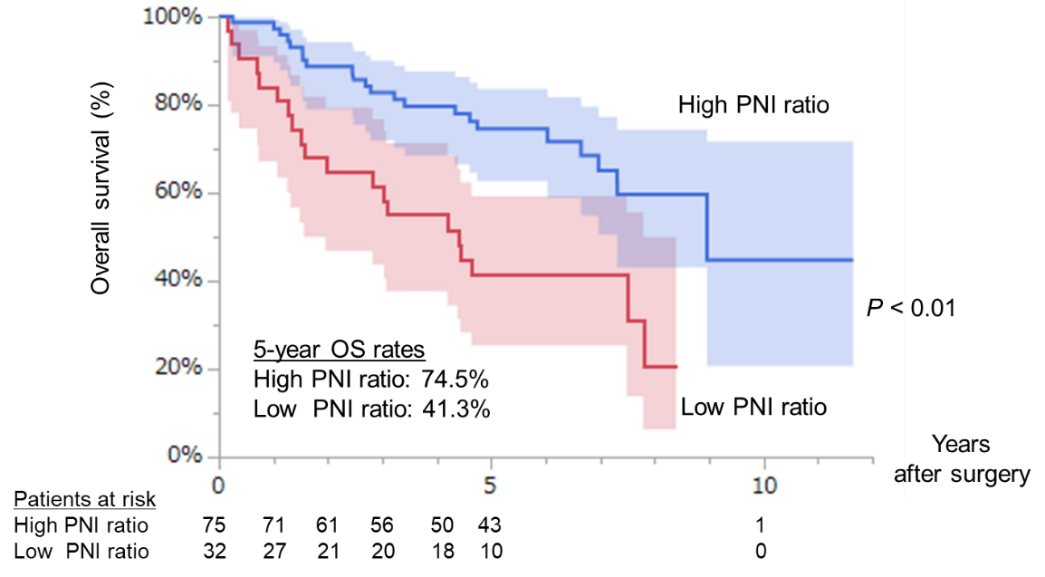


(c)

**
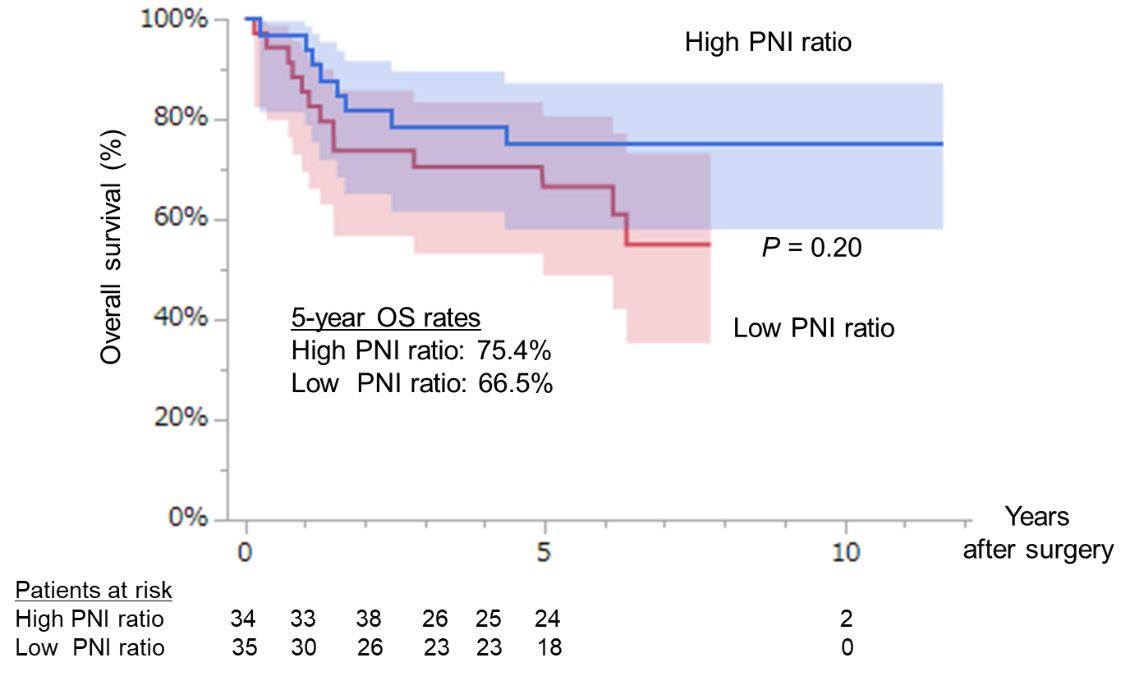
**

(d)


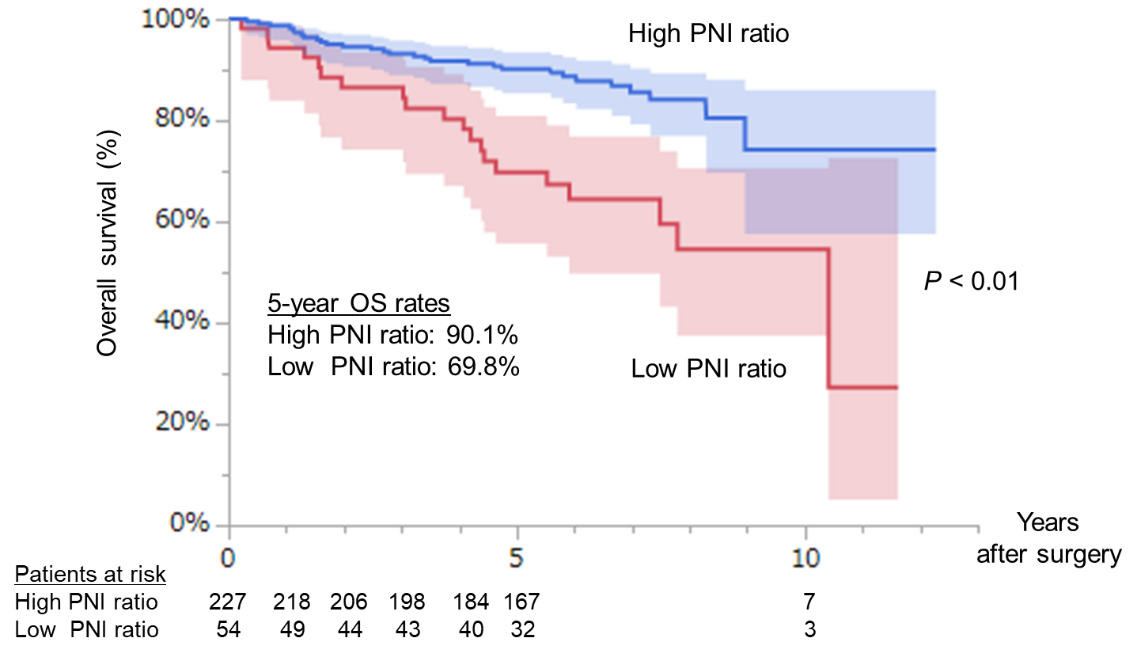


**Supplemental Figure 3.** Receiver operating characteristic curves were used to determine the optimal cut-off values of the (a) preoperative prognostic nutritional index (pre-PNI) and (b) postoperative PNI (post-PNI) for overall survival. AUC: Area under the curve


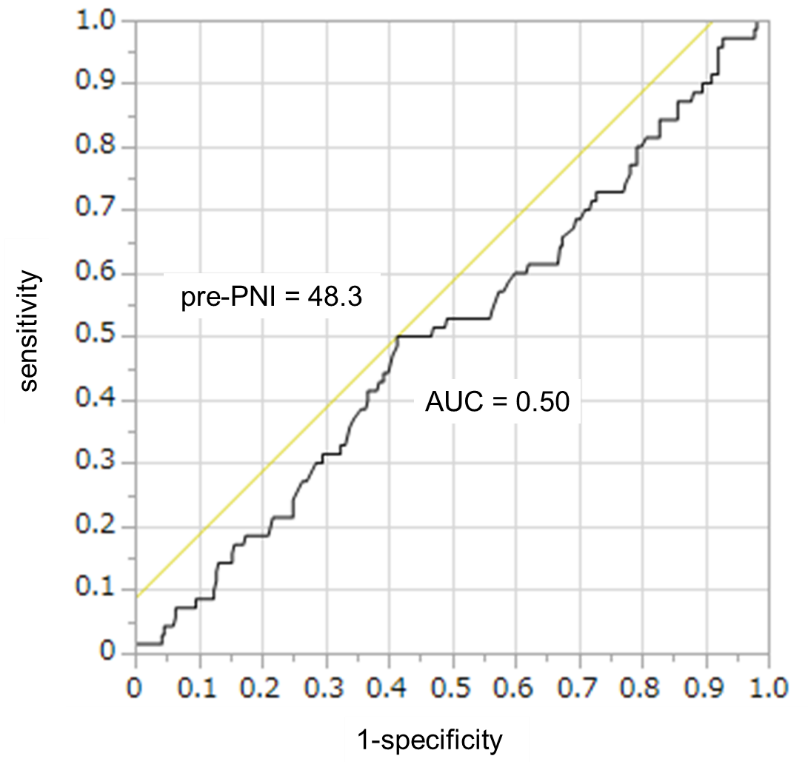


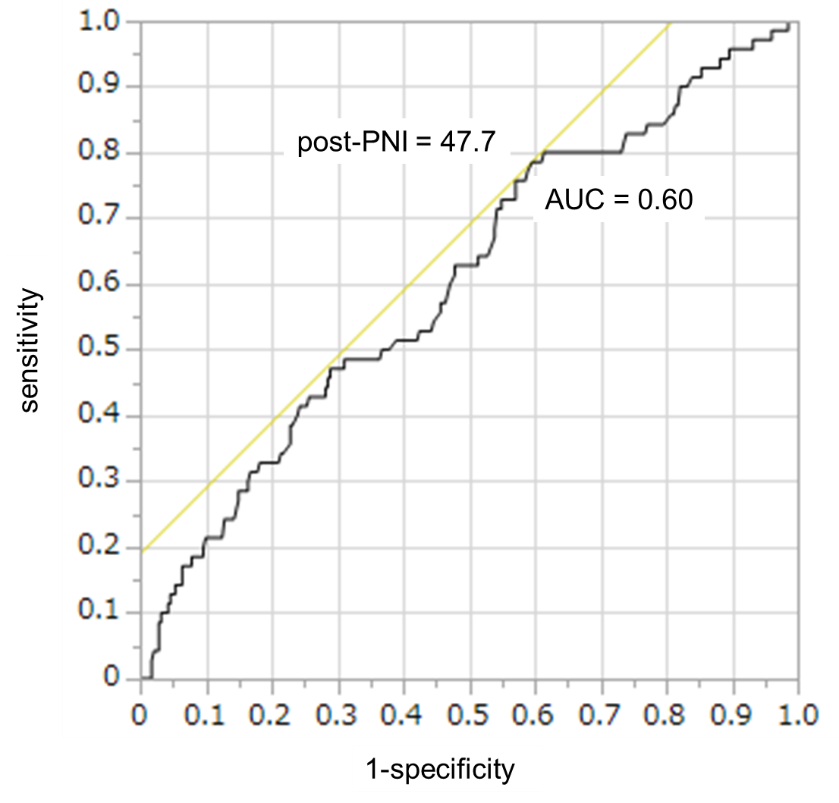

Supplement: Supplementary file 2 — Supplementary file2 (DOCX 918 KB) [file 595_2024_2847_MOESM2_ESM.docx]
